# From Evaluation to Implementation: A Delphi Consensus Framework with Domain-Weighted Decision Models for Robotic Surgery Adoption

**DOI:** 10.3390/healthcare14162637

**Published:** 2026-08-20

**Authors:** Kenneth Chen, Tsi Yu Poon, Wai Chye Cheong, May Anne Cheong, Yvonne Ying Ru Ng, Alvin Yuanming Lee, Ye Xin Koh, Meihuan Chang, Kenny Wei-Tsen Loh, Christine Gaik Yie Neoh, Shirlena Tieu Kwee Wong, Pin Sun Chang, Shu Hui Yeang, An Lui Wang, Kelvin Ghim Chuan Tan, Wai Ching Lee, Jeremy Chon Wai Aw, Carol Siow Yen Ang, Emile John Kwong Wei Tan, Brian Kim Poh Goh, Song Tar Toh, John Shyi Peng Yuen, Henry Sun Sien Ho

**Affiliations:** 1 SGH Department of Urological Surgery, Singapore General Hospital, Singapore 169608, Singapore; 2 SingHealth Alice Lee Innovation Centre of Excellence, SingHealth Group, Singapore 168582, Singapore; 3 SGH Department of Colorectal Surgery, Singapore General Hospital, Singapore 169608, Singapore; 4 SGH Department of Hepatopancreatobiliary & Transplant Surgery, Singapore General Hospital, Singapore 169608, Singapore; 5 SGH Department of Anaesthesiology, Singapore General Hospital, Singapore 169608, Singapore; 6 SGH Medical Board, Singapore General Hospital, Singapore 169608, Singapore; 7 SGH Operating Theatres, Singapore General Hospital, Singapore 169608, Singapore; 8 SGH Office of Patient Safety and Quality, Singapore General Hospital, Singapore 169608, Singapore; 9 SGH Bio-Medical Engineering, Singapore General Hospital, Singapore 169608, Singapore; 10 ALPS-CGH Non-Pharma Supply Chain, SingHealth Group, Singapore 168582, Singapore; 11 SGH Finance & Accounting, Singapore General Hospital, Singapore 169608, Singapore; 12 SGH Facilities Management & Engineering, Singapore General Hospital, Singapore 169608, Singapore; 13 SingHealth Group Communications, SingHealth Group, Singapore 168582, Singapore; 14 SGH Department of Otorhinolaryngology–Head & Neck Surgery, Singapore General Hospital, Singapore 169608, Singapore

**Keywords:** surgical robotics, modified Delphi, implementation framework, hospital leadership, multidisciplinary consensus, patient safety, health technology assessment, governance, sustainability

## Abstract

**Highlights:**

**What are the main findings?**

- A comprehensive, multidisciplinary framework comprising 417 consensus-derived items across nine thematic domains was developed, providing a structured approach for leadership teams to evaluate, assess readiness for, and implement surgical robotic systems.
- The framework integrates diverse stakeholder perspectives through domain-weighted scoring models (clinician, safety, and procurement lenses), enabling balanced and context-specific decision-making for robotic surgery adoption and governance.

**What are the implications of the main findings?**

- Hospitals can use the framework to systematically identify risks, readiness gaps, and implementation requirements, supporting safer and more sustainable adoption of surgical robotic systems.
- The framework promotes informed, transparent, and stakeholder-aligned decision-making, helping healthcare organizations balance clinical value, patient safety, operational feasibility, and long-term strategic investment when expanding robotic surgery programs.

**Abstract:**

**Background:** Adoption of surgical robotic systems is accelerating globally, driven by advances in clinical capability, ergonomics, and workflow efficiency. However, robotic surgery programs introduce complex organizational challenges related to patient safety, workforce capability, infrastructure readiness, governance, vendor dependency, procurement, and long-term sustainability. Hospitals often lack structured, leadership-oriented frameworks to support systematic, context-appropriate evaluation, adoption, and governance of these technologies. **Objective:** To develop a multidisciplinary, consensus-based framework to support leadership-level evaluation, organizational readiness assessment, adoption, and governance of surgical robotic systems. **Methods:** A two-round modified Delphi study was conducted at Singapore General Hospital involving eleven stakeholder groups spanning clinical, perioperative, technical, operational, governance, and executive domains. Department-level consensus responses were generated. In Round 1, stakeholders completed role-specific questionnaires comprising Likert-scale items and open-ended questions. Items rated as low importance were excluded, while items of moderate importance were refined through thematic analysis. In Round 2, refined and newly generated items were re-evaluated. Items achieving ≥70% agreement for high importance within each stakeholder group were retained. **Results:** A total of 417 consensus-derived items were identified and organized into nine thematic domains encompassing patient safety and risk management, technical capability and surgical performance, workflow integration, training and credentialing, governance and sustainability, vendor support, infrastructure, logistics, and operational readiness. The framework was operationalized through category-specific interpretive scoring rubrics and three domain-weighted decision models reflecting clinician, safety, and procurement perspectives. **Conclusions:** This study presents a leadership-oriented, multi-stakeholder framework to support the risk-aware evaluation, adoption, implementation, and governance of surgical robotic systems. By integrating considerations across institutional readiness, comparative robotic platform assessment, and program governance, the framework provides a structured approach to decision-making throughout the robotic surgery lifecycle. While it may serve as a useful starting point for institutions seeking to establish or expand robotic surgery programs, the framework was developed and evaluated within a single academic tertiary healthcare institution. Further validation across diverse healthcare settings, including community hospitals, non-academic institutions, specialty centers, and resource-constrained environments, is required before broader adoption can be recommended.

## 1. Introduction

Surgical robotic systems have become an increasingly important component of contemporary surgical practice, with rapid adoption across tertiary academic medical centers worldwide. These platforms offer potential advantages in technical precision, visualization, dexterity, and surgeon ergonomics. As robotic surgery programs mature, however, robotic systems are increasingly recognized not merely as clinical technologies but as major organizational investments with implications that extend beyond the operating room.

Successful implementation requires considerably more than technology acquisition. Hospitals must address challenges related to workforce training and credentialing, operating room workflows, infrastructure readiness, sterile processing, supply chain management, interoperability, vendor dependence, maintenance planning, financial sustainability, governance, and patient safety oversight. Robotic surgery is therefore best viewed as a complex sociotechnical intervention that must be integrated into existing organizational systems rather than as a standalone technological upgrade.

Despite the rapid growth of robotic-assisted surgery, much of the literature focuses on clinical outcomes, procedural performance, learning curves, and economic evaluations, with comparatively limited attention given to the organizational processes that underpin successful implementation, scale-up, and long-term sustainability. In practice, decisions regarding robotic surgery involve a broad range of stakeholders, including surgeons, anesthesiologists, perioperative nurses, sterile processing personnel, biomedical engineers, facilities managers, procurement and finance professionals, patient safety specialists, and medicolegal advisors. Consequently, adoption decisions are inherently multidisciplinary and depend as much on organizational governance and operational readiness as on technical or clinical considerations.

Several frameworks have been proposed to support robotic surgery evaluation, implementation, and governance; however, most are prescriptive, confined to specific stages of adoption, or focused on individual domains rather than the broader institutional context [1,2,3,4,5,6]. Many treat evaluation, adoption, and governance as separate activities rather than interconnected organizational processes [1,5]. Others emphasize high-level principles while providing limited guidance on how robotic programs are operationalized within real-world healthcare systems [6]. Although stakeholder engagement is increasingly recognized as important, existing approaches often prioritize individual expert opinions over departmental or institutional perspectives, despite major technology decisions typically being governed through organizational structures and multidisciplinary committees [2,3,4]. As a result, important operational, governance, safety, and sustainability considerations may be insufficiently represented during planning and decision-making.

Singapore General Hospital (SGH), a large tertiary academic medical center with extensive robotic surgery experience, provides a unique setting in which to examine these challenges. Following the adoption of the da Vinci Surgical System (Intuitive Surgical, Sunnyvale, CA, USA) and subsequent implementation of the hinotori Surgical Robot System (Medicaroid Corporation, Kobe, Japan), SGH became Singapore’s first multi-platform robotic surgery center. This experience highlighted the extent to which successful robotic surgery programs depend on institutional governance, multidisciplinary coordination, and organizational readiness, rather than solely on clinician preference or technical performance.

There remains a need for practical, leadership-oriented frameworks that support decision-making across the entire robotic surgery lifecycle, from evaluation and procurement to implementation, expansion, and ongoing governance. Such frameworks should incorporate perspectives from the diverse stakeholder groups involved in robotic surgery while providing a structured approach to assessing organizational readiness, implementation requirements, and institutional priorities. A department-level Delphi methodology offers a mechanism for capturing institution-wide consensus that reflects the collective judgment of stakeholder groups rather than isolated individual opinions. When combined with domain-weighted evaluation models, this approach moves beyond traditional checklist-based assessments to provide a more nuanced understanding of organizational readiness and strategic priorities.

Accordingly, this study aimed to develop a leadership-oriented, multidisciplinary framework to support the evaluation, adoption, implementation, and governance of surgical robotic systems within complex healthcare organizations. Designed for both first-time adopters and institutions seeking to expand or diversify existing robotic surgery programs, the framework supports decision-making across the robotic surgery lifecycle, including pre-procurement assessment, implementation planning, and post-implementation governance. By integrating institutional perspectives across clinical, operational, technical, governance, financial, and patient safety domains, the framework provides a structured, risk-aware, and context-sensitive approach to robotic surgery adoption that is aligned with broader organizational priorities.

## 2. Methods

### 2.1. Study Setting, Design, and Rationale

This study was conducted at SGH, a high-volume tertiary academic medical center with established robotic surgery programs, complex perioperative workflows, and mature governance structures. The institution provided a real-world environment for examining organizational factors that influence the adoption, implementation, and governance of robotic surgery.

**Figure 1:**
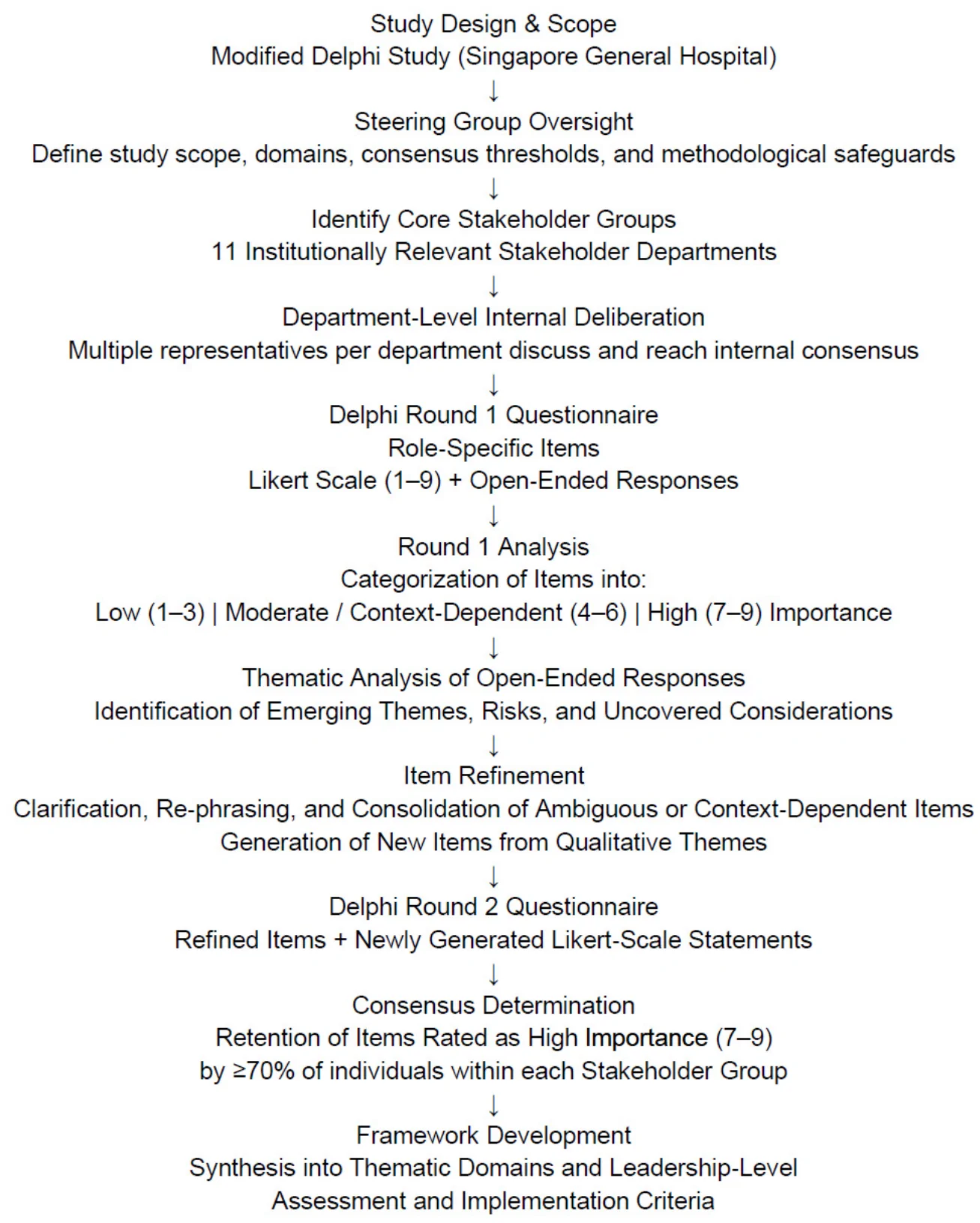
Modified Delphi process underpinning framework development. Department-level consensus across 11 stakeholder groups informed framework construction through combined quantitative and qualitative analysis.

A two-round modified Delphi methodology was used to establish consensus on factors considered important for robotic surgery adoption and implementation (Figure 1). Unlike conventional Delphi studies that rely on individual expert opinions, this study was designed to reflect how major healthcare technology decisions are made in practice, where adoption and investment decisions are typically shaped through departmental deliberation and committee-based governance.

Accordingly, the Delphi process was conducted at the departmental level. Each stakeholder group deliberated internally and submitted a single consolidated response for each round. This approach was intended to capture collective professional judgment, minimize the influence of individual preferences, and enhance the institutional relevance of the resulting framework.

Participants were recruited from departments directly involved in the evaluation, procurement, implementation, governance, and operational use of robotic surgical systems at SGH. Eligible participants included individuals with direct or indirect responsibility for clinical, operational, administrative, financial, technical, or governance functions related to robotic surgery programs. Recruitment was purposively focused on stakeholders with institutional experience derived from SGH’s implementation of both the da Vinci Surgical System and the hinotori Surgical Robot System.

### 2.2. Stakeholder Identification and Recruitment

Stakeholder mapping initially identified seventeen departments involved in robotic surgery planning, delivery, support, or governance. Following review to reduce redundancy while preserving distinct functional perspectives, eleven stakeholder groups were included: robotic surgeons, anesthesiologists, perioperative nurses, sterile services personnel, biomedical engineers, facilities and infrastructure teams, patient safety, quality and experience leaders, finance representatives, procurement leaders, legal and medical risk management representatives, and marketing and communications leaders.

Roles with substantial functional overlap were consolidated. Operating room technicians and cybersecurity personnel were represented by the biomedical engineering group; scrub and circulating nurses were combined into a single perioperative nursing group; and overlapping leadership responsibilities were represented by their respective stakeholder groups. Individuals with declared conflicts of interest were excluded. Participation was voluntary and uncompensated.

A detailed description of participant composition, stakeholder representation, and seniority distribution is provided in Supplementary Section S1.

### 2.3. Questionnaire Development

Questionnaires were developed through a structured, multistage process. During the exploratory phase, artificial intelligence-assisted tools (Microsoft Copilot 2026) were used to support the synthesis and organization of an initial item pool spanning clinical, technical, operational, governance, and implementation domains. Source materials included institutional planning documents, evaluation criteria, governance workflows, multidisciplinary meeting records, and implementation experiences associated with SGH’s robotic surgery programs.

AI-assisted methods were used solely for information organization and thematic synthesis. They were not used to define framework domains, establish evaluation criteria, determine methodology, generate findings, or formulate study conclusions. All outputs underwent independent review, verification, and refinement by the study team before inclusion in the study.

Draft items were subsequently reviewed by a multidisciplinary steering panel comprising surgeons, anesthesiologists, perioperative nursing leaders, biomedical engineers, patient safety representatives, health technology assessment specialists, and legal advisors. Items were revised or removed based on clarity, redundancy, contextual relevance, and institutional applicability.

**Figure 2:**
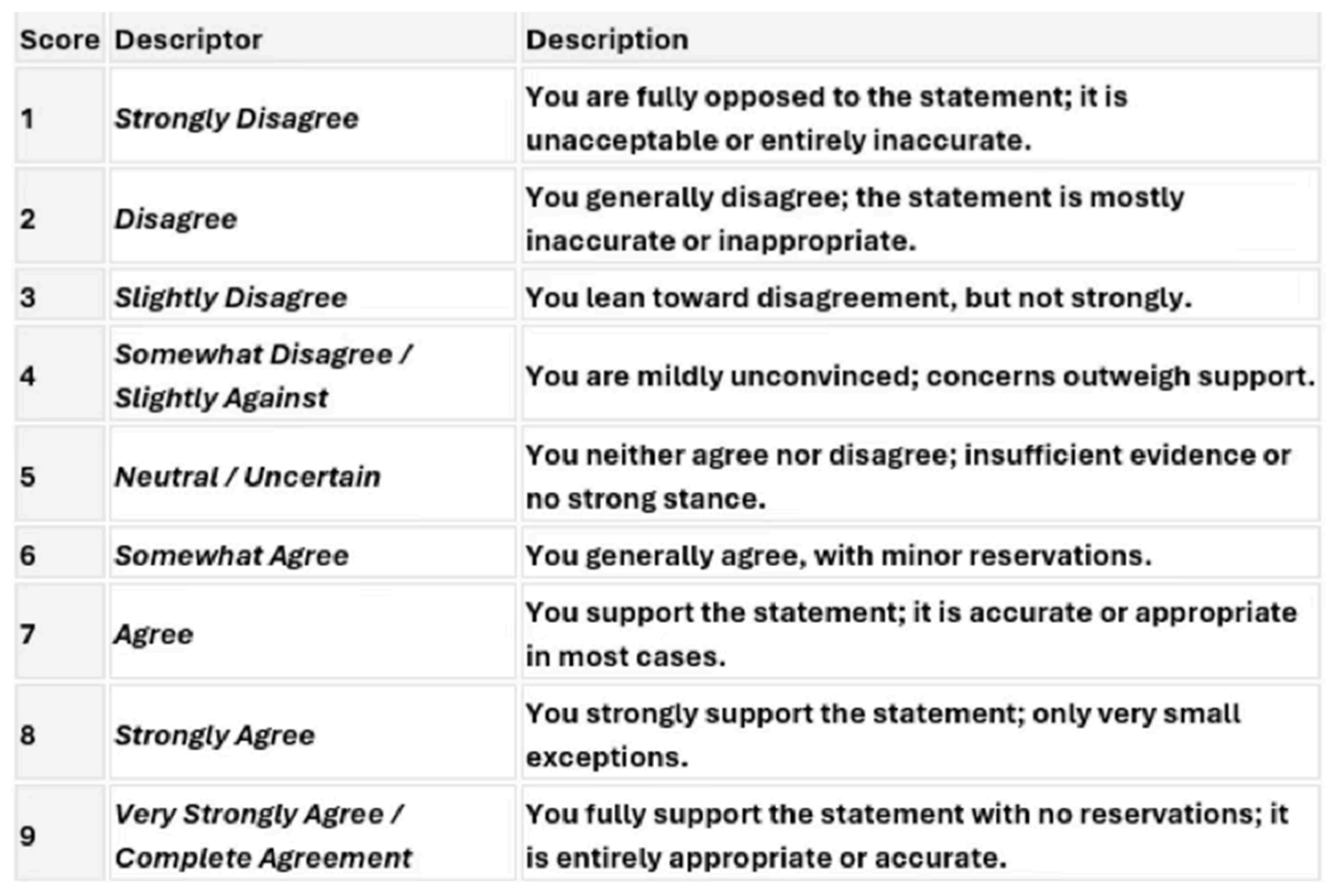
Nine-point Likert scale used in the modified Delphi process. Items were rated by perceived importance using predefined anchors—low (1–3), moderate or context-dependent (4–6), and high (7–9)—to inform item prioritization, refinement, and consensus determination.

Role-specific questionnaires were then developed for each stakeholder group. Each questionnaire comprised closed-ended items rated on a nine-point Likert scale (1–3, low importance; 4–6, moderate or context-dependent importance; 7–9, high importance), together with open-ended questions to capture additional considerations (Figure 2).

### 2.4. Modified Delphi Rounds and Consensus Determination

Rounds 1 and 2 were administered electronically using FormSG, a secure government platform. Stakeholder groups reviewed items collaboratively and submitted a single consolidated departmental response, which served as the unit of analysis.

Within each stakeholder group, participants independently assessed candidate items before engaging in structured discussions facilitated by a designated departmental representative. These discussions explored areas of agreement, disagreement, and differing interpretations to establish a shared departmental position.

Consensus was defined a priori as ≥70% agreement within a stakeholder group and was operationalized as at least 70% of participants assigning an item to the same predefined importance category (low: 1–3, moderate: 4–6, or high: 7–9), irrespective of the specific score selected within that category. Mean and median scores were not used as primary measures of consensus because they may mask meaningful disagreement within stakeholder groups. Instead, consensus was determined based on the proportion of participants assigning an item to the same importance category. Where disagreement persisted, facilitated discussions were used to clarify interpretations and identify sources of divergence. A consolidated departmental response was recorded only after the predefined consensus threshold had been achieved. All stakeholder groups ultimately reached the required level of consensus.

Following Round 1, items rated as low importance (1–3) were excluded. Items rated as high importance (7–9) that achieved consensus were provisionally retained and were not re-evaluated in subsequent rounds. Items that did not meet the predefined consensus threshold were classified as non-consensus items and subjected to further review and refinement before reassessment. This approach reduced participant burden while focusing subsequent rounds on areas of uncertainty or disagreement.

Items receiving moderate importance ratings (4–6) underwent structured review before Round 2. The study team sought clarification and qualitative feedback from relevant stakeholder groups to better understand the reasons underlying these ratings. Common issues included ambiguous terminology, context-dependent interpretations, and statements containing multiple concepts that stakeholders considered distinct.

Consistent with established Delphi methodology, items were refined through clarification of terminology, revision of definitions, neutralization of prescriptive language, restructuring of item wording, and separation of compound concepts where appropriate (Supplementary Section S6). During Round 2, participants reviewed aggregated feedback from Round 1 and re-evaluated revised items. This iterative process facilitated convergence toward consensus while preserving the underlying concepts being assessed.

Open-ended responses were analyzed using a hybrid inductive-deductive thematic approach [7,8,9,10,11,12]. Inductive coding identified emergent risks, operational challenges, and contextual dependencies, while deductive categorization drew upon established frameworks relating to surgical innovation, health technology assessment, enterprise risk management, and healthcare governance [13,14,15,16,17,18,19,20]. Emerging themes informed the refinement of existing items and the development of new items for Round 2.

### 2.5. Data Management, Bias Mitigation, and Oversight

Several measures were implemented to minimize potential sources of bias. Cross-group discussions were not permitted, individual responses were not disclosed, and only anonymized aggregated feedback was shared between Delphi rounds. Questionnaires were vendor-neutral and excluded proprietary performance claims or commercially identifiable content.

Quantitative data were analyzed descriptively at the departmental level using Microsoft Excel. Qualitative data were organized using structured coding matrices according to domain, stakeholder group, and theme and were subsequently integrated with quantitative findings [21].

The study received approval from the SingHealth Centralized Institutional Review Board. Written informed consent was obtained from all participants (Supplementary Section S3). Oversight was provided by a multidisciplinary steering group responsible for methodological governance, adjudication of borderline items, and ensuring balanced stakeholder representation. Steering group members did not participate as Delphi respondents. Conflicts of interest were declared and managed in accordance with institutional procedures (Supplementary Section S4).

### 2.6. Framework Development and Ranking Exercises

Consensus-derived items were synthesized into nine thematic domains representing key organizational considerations relevant to robotic surgery adoption, implementation, and governance. Domains were independently reviewed by the investigators for conceptual coherence and mapped to institutional decision-making processes.

To accommodate different organizational perspectives, three domain-weighted models were developed: a clinician-weighted model, a patient safety-weighted model, and a procurement-weighted model. Weightings were developed using a normative, literature-informed approach intended to reflect differing institutional priorities, responsibilities, and accountability structures.

Stakeholder groups subsequently completed forced-ranking exercises to rank both the relative importance of the nine domains and the influence of stakeholder groups on robotic surgery adoption decisions. These exercises were designed to reveal explicit trade-offs and identify areas of convergence and divergence in perceived institutional priorities, influence, and accountability.

## 3. Results

### 3.1. Consensus-Based Item Development

Each stakeholder group reviewed questionnaire items collaboratively and submitted a single consensus response, resulting in an institution-level dataset comprising 417 consensus-derived items. The dataset reflects collective departmental judgments across participating stakeholder groups rather than aggregated individual opinions. The complete 417-item instrument is available from the corresponding author upon request.

### 3.2. Identification of Thematic Domains

Thematic analysis of the consensus-derived items identified nine overarching domains representing key organizational considerations for robotic surgery adoption, implementation, and governance (Table 1). Domains were derived through iterative clustering based on conceptual similarity, relevance to robotic surgery programs, and alignment with institutional decision-making processes.

The nine domains were: (1) Patient Safety & Risk Management; Emergency Preparedness & Crisis Management; (2) Technical Capability & Surgical Performance; (3) Intraoperative Workflow & Operational Integration; (4) Training, Skill Acquisition & Trainee Governance; (5) Credentialing, Supervision & Educational Infrastructure; (6) Evaluation, Strategic Governance, Procurement, Adoption & Sustainability; (7) Vendor & Industry Support; Vendor Performance & Transparency; (8) Operating Room Setup, Layout & Environmental Fit; and (9) Supplies, Logistics & Turnover.

### 3.3. Domain-Weighted Priority Models

Three domain-weighted models were developed to reflect common organizational perspectives encountered during robotic surgery evaluation, procurement, implementation, and governance: clinician-weighted, patient safety-weighted, and procurement-weighted models (Table 2 and Table 3). Rather than assuming a single universal prioritization framework, these models recognize that institutional priorities vary according to governance structures, regulatory obligations, financial constraints, stakeholder composition, and organizational risk tolerance.

The weightings were derived using a normative, literature-informed approach. Domain prioritization was informed by published evidence relating to surgical performance, patient safety, organizational governance, health technology assessment, and technology procurement, rather than statistical optimization. The aim was to develop illustrative decision-support models representing three common institutional viewpoints: clinical performance, patient safety and risk management, and organizational stewardship. Further details are provided in Supplementary Section S2.

The clinician-weighted model places greater emphasis on Technical Capability & Surgical Performance, Intraoperative Workflow & Operational Integration, Training, Skill Acquisition & Trainee Governance, and Credentialing, Supervision & Educational Infrastructure, reflecting priorities related to usability, procedural performance, operational efficiency, and workforce development [22,23,24]. The patient safety-weighted model prioritizes Patient Safety & Risk Management; Emergency Preparedness & Crisis Management and Evaluation, Strategic Governance, Procurement, Adoption & Sustainability, reflecting concerns related to harm prevention, regulatory compliance, and institutional accountability [25,26]. The procurement-weighted model places greater emphasis on strategic governance, sustainability, vendor performance, lifecycle costs, scalability, and exit flexibility, reflecting an enterprise stewardship perspective focused on long-term organizational value and risk management [27,28,29,30].

All three models retain the same nine-domain structure, differing only in the relative weight assigned to each domain. Together, they illustrate how a common evaluation framework can be viewed through clinical, safety, and organizational lenses while preserving a consistent underlying architecture.

### 3.4. Leadership-Focused Framework and Scoring Rubrics

Integration of the consensus-derived items, thematic domains, and domain-weighted models resulted in a leadership-oriented framework designed to support robotic surgery evaluation, organizational readiness assessment, technology selection, implementation planning, and ongoing program governance.

The final framework comprises 417 items organized into three complementary categories addressing distinct decision-making needs: Category 1: Institutional Readiness Assessment; Category 2: Comparative Platform Evaluation; and Category 3: Governance and Implementation Requirements (Table 4). Collectively, these categories support decision-making across the robotic surgery lifecycle, from pre-procurement assessment and platform selection to implementation, oversight, and continuous program evaluation.

Category 1 evaluates an organization’s capacity to adopt and sustain a robotic surgery program, encompassing governance structures, workforce capability, infrastructure readiness, operational preparedness, training capacity, and patient safety processes. Category 2 supports the comparative assessment of robotic platforms by evaluating platform-specific characteristics such as technical performance, safety features, usability, workflow integration, ergonomics, functionality, and related system capabilities. Category 3 focuses on the governance structures, policies, accountability mechanisms, operational safeguards, and oversight processes required to support safe, effective, and sustainable robotic surgery programs regardless of the platform selected.

The framework incorporates item-level scoring, domain-level aggregation, and application of the selected weighting model to generate composite scores aligned with different institutional perspectives. Category-specific scoring rubrics are provided in Table 5, Table 6 and Table 7, covering Institutional Readiness Assessment, Comparative Platform Evaluation, and Governance and Implementation Performance. These rubrics support consistent interpretation and application of the framework across organizational, procurement, implementation, and governance contexts. A worked example is provided in Supplementary Section S5.

Composite score thresholds (Table 5, Table 6 and Table 7) were developed using established principles from health technology assessment, risk governance, and organizational maturity frameworks [28,31,32,33,34,35,36,37,38,39,40,41]. As these thresholds are based on expert consensus and normative judgments rather than empirical optimization, they should be interpreted as pragmatic decision-support guides.

### 3.5. Domain and Stakeholder Importance Rankings

Domain ranking exercises demonstrated a consistent emphasis on patient safety, clinical performance, and operational effectiveness across stakeholder groups (Table 8). Domain 1 (Patient Safety & Risk Management; Emergency Preparedness & Crisis Management) achieved the lowest total ranking score and was therefore identified as the most important domain overall (total score = 17). This was followed by Domain 2 (Technical Capability & Surgical Performance; total score = 23) and Domain 3 (Intraoperative Workflow & Operational Integration; total score = 37).

Domains related to training, credentialing, and governance occupied intermediate positions, with total scores ranging from 50 to 60. Domains addressing vendor-related, infrastructure-related, and logistics-related considerations received comparatively higher scores (66–78), indicating lower relative priority within the ranking exercise.

Stakeholder ranking exercises similarly demonstrated broad agreement regarding the relative influence of different stakeholder groups in robotic surgery adoption decisions (Table 9). Surgeons were ranked as the most influential stakeholder group overall (total score = 17), followed by Patient Safety, Quality and Experience representatives (43), perioperative nurses (44), and anesthesiologists (46). Biomedical engineering, infrastructure, and sterile services groups occupied intermediate positions, while finance, procurement, senior leadership, and marketing and communications groups received progressively higher scores.

Overall, the ranking exercises demonstrated strong alignment across stakeholder groups regarding the central importance of patient safety, clinical performance, and frontline clinical stakeholders in robotic surgery adoption, implementation, and governance.

## 4. Discussion

### 4.1. Principal Findings and Contribution

This study presents a leadership-oriented framework for the evaluation, adoption, implementation, and governance of surgical robotic systems, developed using a modified Delphi methodology designed to reflect real-world hospital decision-making. By engaging eleven stakeholder groups spanning clinical, perioperative, technical, operational, governance, and administrative functions, the framework addresses an important limitation of existing approaches, which often emphasize clinical or economic outcomes while underrepresenting the broader organizational factors that influence successful technology adoption.

A key contribution of this study is its recognition that adoption of high-impact healthcare technologies is inherently an organizational rather than individual process. Conducting the Delphi process at the departmental level mirrors how major hospital decisions are typically made, through collective deliberation within functional groups and governance structures that balance clinical objectives, operational feasibility, financial considerations, and organizational risk. This alignment between methodology and practice enhances both the institutional relevance and practical applicability of the framework.

The identification of 417 consensus-derived items reinforces the view that robotic surgery implementation is fundamentally a sociotechnical undertaking. Stakeholders consistently emphasized patient safety, workforce readiness, infrastructure capacity, governance oversight, vendor dependency, and long-term sustainability as critical determinants of program success. Concerns relating to emergency preparedness, training capacity, medicolegal risk, and lifecycle management further suggest that narrowly focused assessments may overlook challenges that emerge during implementation and ongoing operations. The inclusion of open-ended responses provided additional insight into contextual dependencies and latent organizational risks that may not be captured through structured scoring approaches alone.

The proposed framework complements existing approaches such as IDEAL and hospital-based HTA by operationalizing their underlying principles within the context of robotic surgery governance. By translating high-level implementation concepts into actionable organizational considerations, it provides a structured mechanism for assessing readiness, informing implementation planning, and supporting continuous program evaluation. In doing so, it helps bridge the gap between conceptual recommendations and the practical realities of robotic surgery deployment within healthcare organizations.

### 4.2. Value of Thematic Domain Structuring

Organizing the 417 consensus-derived items into nine thematic domains transformed a complex body of stakeholder input into a practical and accessible decision-support framework. Collectively, the domains encompass the principal organizational dimensions of robotic surgery adoption, including clinical performance, patient safety, workflow integration, workforce development, governance, infrastructure, vendor management, and logistics.

Importantly, many domains incorporated contributions from multiple stakeholder groups, highlighting that responsibility for robotic surgery adoption and oversight is distributed across the organization rather than confined to individual professions or departments. This finding underscores the importance of integrated governance structures that promote collaboration, align priorities, and coordinate accountability across traditionally siloed functions.

### 4.3. Interpretation of Domain-Weighted Models

The clinician-weighted, patient safety-weighted, and procurement-weighted models illustrate how robotic surgery programs may be evaluated through different organizational perspectives. Developed using a normative, literature-informed methodology, these models are intended as decision-support tools rather than empirically optimized scoring systems. Their purpose is not to prescribe a single prioritization framework but to acknowledge that institutional priorities vary according to governance structures, regulatory requirements, financial constraints, stakeholder composition, program maturity, and organizational risk tolerance.

The clinician-weighted model emphasizes technical capability, surgical performance, workflow integration, and training, reflecting frontline priorities related to procedural effectiveness and user experience. In contrast, the patient safety-weighted model prioritizes harm prevention, emergency preparedness, governance, and risk management. The procurement-weighted model adopts an enterprise stewardship perspective that emphasizes governance, sustainability, vendor performance, lifecycle costs, scalability, and exit flexibility.

Together, these models demonstrate how a shared domain architecture can be adapted to support different organizational objectives while maintaining a consistent evaluative framework. Accordingly, the proposed weightings should be viewed as illustrative representations of common institutional perspectives rather than universal standards. Alternative weighting arrangements may be equally appropriate in different healthcare settings. Future multicenter studies may help evaluate alternative weighting approaches and explore empirical optimization methods to further strengthen the framework’s validity, applicability, and generalizability.

### 4.4. Leadership Framework and Interpretive Utility

The integration of consensus-derived items, thematic domains, and domain-weighted models resulted in a leadership-oriented framework capable of translating detailed stakeholder input into actionable organizational insights. A central design principle is the separation of content, weighting, and interpretation, enabling institutions to apply a common framework structure while tailoring evaluation priorities to local governance objectives and strategic needs. Consequently, composite scores reflect alignment with a selected institutional perspective rather than an absolute measure of technological merit.

The accompanying interpretive rubric translates quantitative outputs into decision-relevant categories while reducing the risk that strengths in some areas might obscure critical deficiencies in others. This approach is consistent with principles established in health technology assessment and risk governance frameworks [42,43,44,45], supporting context-sensitive and conditional decision-making [36,46,47]. The framework is intended to inform leadership deliberations rather than replace professional judgment, regulatory oversight, or formal technology assessment processes.

Although composite scores provide an overall assessment of readiness, domain-level scores offer additional diagnostic value by identifying specific strengths, weaknesses, and areas requiring remediation. This enables leadership teams to prioritize capability-building efforts, allocate resources more effectively, and develop targeted implementation strategies before program deployment.

### 4.5. Interpretation of Ranking Exercises

The ranking exercises demonstrated strong agreement regarding the importance of patient safety, clinical performance, and operational effectiveness. They also revealed differences in how stakeholder groups perceived influence, accountability, and decision-making authority within robotic surgery programs. These findings highlight the importance of governance structures that are transparent, inclusive, and capable of balancing diverse institutional perspectives.

The rankings presented in Table 8 and Table 9 should be interpreted within the organizational context in which the framework was developed. They reflect the collective perspectives of stakeholders from a large tertiary academic medical center with established robotic surgery programs, mature governance processes, and clearly defined organizational responsibilities. Consequently, the relative importance assigned to individual domains and stakeholder groups may be influenced by local governance arrangements, operational roles, and accumulated institutional experience.

Because responsibilities for robotic surgery procurement, implementation, maintenance, training, quality assurance, and governance vary considerably across healthcare systems, institutions with different organizational structures may prioritize domains differently. Accordingly, the rankings reported in this study should not be interpreted as normative benchmarks.

The framework was intentionally designed to accommodate such variation. While the nine domains provide a consistent multidisciplinary structure, institutions may adapt domain weightings to reflect local governance arrangements, stakeholder responsibilities, strategic priorities, regulatory requirements, and available resources. This flexibility enables context-specific application while preserving a common evaluative framework.

Furthermore, rankings were derived from stakeholder-group consensus rather than individual responses. Because major organizational decisions are generally shaped by collective departmental priorities rather than personal preferences, this approach was considered more representative of institutional decision-making. Nevertheless, the findings should be interpreted as institution-specific observations rather than universally generalizable conclusions. Future multicenter studies may provide insight into how organizational priorities differ across healthcare systems, governance models, and stages of robotic surgery program maturity.

### 4.6. Limitations and Future Directions

Several limitations should be considered when interpreting these findings. First, the framework was developed and evaluated within a single high-volume academic tertiary healthcare institution with established robotic surgery programs, mature governance structures, dedicated educational resources, and comprehensive quality assurance processes. While this setting provided substantial institutional experience across the robotic surgery lifecycle, it may limit the immediate generalizability of the findings to other healthcare environments. Organizational structures, stakeholder priorities, governance arrangements, clinical workflows, resource availability, and procurement processes may differ considerably across institutions and healthcare systems. Consequently, although the framework was intentionally designed to be adaptable and broadly applicable, its transferability to other settings remains to be established. External validation in diverse healthcare environments, including non-tertiary hospitals, community healthcare settings, non-academic institutions, and resource-constrained contexts, will be necessary before broader adoption can be recommended.

Second, some framework items may reflect local operational practices, governance requirements, and organizational priorities arising from SGH’s experience implementing and managing two robotic surgical platforms. The framework should therefore be regarded as a structured guidance tool rather than a prescriptive checklist. The nine thematic domains were intentionally developed to capture broad organizational considerations relevant to robotic surgery evaluation, implementation, and governance while allowing adaptation to local contexts. For smaller hospitals, single-specialty programs, and resource-constrained settings, implementation may require prioritization of selected domains or items, as well as modification of governance processes to align with local capabilities, case volumes, regulatory requirements, and available resources.

Third, several stakeholder groups, particularly Finance (n = 8) and Medical Risk Management & Medicolegal (n = 7), had relatively small sample sizes. As a result, consensus estimates within these groups are more sensitive to the views of individual participants and should be interpreted with appropriate caution. However, the objective of this study was not to generate statistically representative departmental estimates, but rather to obtain multidisciplinary perspectives from stakeholders involved in the adoption, implementation, governance, and operational support of robotic-assisted surgery. Stakeholder groups were selected a priori based on their anticipated roles in robotic surgery programs, and the final framework was informed by convergence across multiple stakeholder categories rather than being driven by any single group’s responses. Nevertheless, department-level consensus may obscure minority viewpoints, representing an inherent trade-off between institutional representation and individual perspectives.

Finally, the domain weightings were normatively derived through stakeholder engagement rather than statistically optimized. While this approach reflects real-world governance and decision-making practices, it introduces the possibility that different stakeholder groups may assign alternative priorities to individual domains [42,48]. To assess the robustness of the framework, deterministic and probabilistic sensitivity analyses were conducted using simulated performance profiles (Supplementary Section S7). These analyses demonstrated that composite score interpretations remained largely stable under reasonable variations in domain weightings, with only solutions positioned near category thresholds showing meaningful sensitivity. Nevertheless, future validation studies using empirical evaluation data will be important to further examine the performance and robustness of the weighting framework in operational settings.

To our knowledge, relatively few studies have proposed comprehensive approaches that integrate institutional readiness assessment, robotic platform evaluation, and program governance within a single framework. We therefore view this work as an exploratory contribution that offers practical insight into how diverse stakeholder groups can be systematically engaged in robotic surgery technology adoption and organizational readiness assessments.

Future research will focus on external and longitudinal validation across institutions with differing levels of robotic surgery experience, governance maturity, organizational structures, and resource availability. Planned studies include regional validation across Southeast Asia and evaluation in a broader range of healthcare contexts, including community hospitals, specialty centers, and resource-constrained settings to assess the framework’s reproducibility, feasibility, and generalizability. Further research will also explore integration of the framework into hospital-based health technology assessment processes to strengthen alignment between stakeholder engagement, readiness assessment, procurement decision-making, and program governance.

## 5. Conclusions

This study developed a multidisciplinary, leadership-oriented framework to support the evaluation, adoption, implementation, and governance of robotic surgery programs. Using a modified Delphi process involving eleven stakeholder groups, 417 consensus-derived items were synthesized into nine thematic domains and organized into three complementary assessment categories addressing institutional readiness, comparative robotic platform evaluation, and governance and implementation requirements. The framework was further operationalized through stakeholder-informed weighting models and category-specific scoring rubrics to support structured decision-making across the robotic surgery lifecycle.

The findings demonstrate that successful robotic surgery adoption extends beyond technology selection and clinical performance. It is a complex organizational endeavor requiring alignment across governance, patient safety, workforce capability, operational readiness, infrastructure, procurement, and long-term sustainability. By integrating perspectives from clinical, operational, safety, engineering, procurement, financial, and administrative stakeholders within a single decision-support framework, this study addresses an important gap in the literature, where existing approaches often focus on isolated aspects of robotic surgery evaluation rather than the broader institutional context.

As robotic surgery platforms continue to proliferate and healthcare organizations face increasing pressure to justify high-value technology investments, structured, transparent, and multidisciplinary decision-making processes are increasingly important. The proposed framework offers a practical approach for assessing organizational readiness, comparing robotic technologies, guiding implementation, and supporting ongoing governance while making institutional priorities and trade-offs explicit.

While the framework may serve as a useful starting point for institutions seeking to establish or expand robotic surgery programs, it was developed and evaluated within a single academic tertiary healthcare institution. Consequently, its broader applicability remains to be established. Further validation across diverse healthcare settings, including community hospitals, non-academic institutions, specialty centers, and resource-constrained environments, is required before broader adoption can be recommended.

Future research should focus on multicenter validation, longitudinal evaluation, and refinement of the proposed weighting models and scoring thresholds across diverse healthcare contexts. Such work will help determine the framework’s generalizability, evaluate its impact on implementation and governance outcomes, and strengthen its utility as a decision-support tool for robotic surgery adoption, evaluation, and oversight.

## Figures and Tables

**Table 1:** Thematic domains and stakeholder mapping for surgical robotic system evaluation. Thematic domains were derived through consensus-based clustering of items generated across stakeholder groups and reflect the distributed nature of organizational responsibility for robotic surgery adoption, implementation, and governance. Domain development emphasized conceptual coherence, minimization of redundancy, and preservation of distinct organizational considerations relevant to institutional decision-making. The resulting framework captures multidisciplinary perspectives across clinical, operational, technical, governance, financial, and patient safety domains. The complete 417-item instrument is available from the corresponding author upon reasonable request.

Thematic Domain	Definition	Key Focus Areas	Primary Stakeholder Groups (Question Numbers)
Domain 1: Patient Safety & Risk Management; Emergency Preparedness & Crisis Management	Addresses the extent to which robotic surgical systems and practices protect patients from preventable harm, including system reliability, emergency preparedness, management of intraoperative complications, and access to technical support to mitigate system related adverse events.	Safety assurance; failure response; emergency readiness; intraoperative risk	Surgeons Round 1 (Q1–7). Surgeons Round 2 (Q3, Q5, Q19, Q21, Q29). Anesthesiologists Round 1 (Q1–14, Q23–26). Anesthesiologists Round 2 (Q1–3). Perioperative nurses Round 1 (Q1–8, Q27–33). Perioperative nurses Round 2 (Q5). Biomedical engineers Round 1 (Q6–9, Q26–28). Biomedical engineers Round 2 (Q6–8, Q19–21). Sterile services unit Round 1 (Q1–8). Sterile services unit Round 2 (Q2–3). Patient safety, quality and experience Round 1 (Q23–28). Procurement Round 1 (Q15).
Domain 2: Technical Capability & Surgical Performance	Captures whether the robotic system provides the technical performance required for safe and effective execution of intended surgical tasks, including dexterity, visualization, responsiveness, ergonomics, and procedural precision.	System functionality; surgeon control; technical adequacy	Surgeons Round 1 (Q8–13). Surgeons Round 2 (Q2, Q14, Q20). Biomedical engineers Round 1 (Q1–5). Biomedical engineers Round 2 (Q14).
Domain 3: Intraoperative Workflow & Operational Integration	Evaluates how well robotic surgery integrates into existing clinical workflows, team communication, and perioperative processes without introducing inefficiencies or disruption.	Usability; team coordination; efficiency; operating room flow	Surgeons Round 1 (Q14–15, Q17–18). Surgeons Round 2 (Q13). Anesthesiologists Round 1 (Q15–21). Perioperative nurses Round 1 (Q9–16). Perioperative nurses Round 2 (Q1–2). Biomedical engineers Round 1 (Q17–20). Biomedical engineers Round 2 (Q12–13). Sterile services unit Round 1 (Q9–14, Q16–17, Q19). Patient safety, quality and experience Round 1 (Q19–21). Procurement Round 1 (Q8, Q13).
Domain 4: Training, Skill Acquisition & Trainee Governance	Focuses on structured training pathways, skill assessment, trainee progression, and oversight mechanisms required to ensure safe and effective role specific development in robotic surgery.	Education structure; competency assessment; trainee safety	Surgeons Round 1 (Q20–28). Surgeons Round 2 (Q1, Q12, Q17–18, Q33–35, Q41). Perioperative nurses Round 2 (Q3). Biomedical engineers Round 2 (Q17–18). Procurement Round 1 (Q10).
Domain 5: Credentialing, Supervision & Educational Infrastructure	Addresses formal systems governing role specific credentialing, authorization, supervision, performance monitoring, and educational support required to sustain safe robotic practice.	Credentialing; supervision; mentoring; learning systems; competency assurance; professional accountability	Surgeons Round 1 (Q29–34). Surgeons Round 2 (Q7–11, Q25–28, Q36–38, Q42–43). Medical risk-management/medicolegal Round 1 (Q29–30). Procurement Round 1 (Q14).
Domain 6: Evaluation, Strategic Governance, Procurement, Adoption & Sustainability	Examines how robotic systems are evaluated, selected, implemented, and sustained at the institutional level, including governance, regulatory, cybersecurity, lifecycle, scalability, and long-term value considerations.	Governance; procurement; system value; program sustainability; change management	Surgeons Round 1 (Q35–40). Surgeons Round 2 (Q4, Q6, Q15–16, Q22–23, Q30–31, Q39–40, Q45). Anesthesiologists Round 1 (Q27, Q29–33). Perioperative nurses Round 1 (Q39–40). Perioperative nurses Round 2 (Q6). Biomedical engineers Round 1 (Q29–30). Biomedical engineers Round 2 (Q1, Q3, Q16). Sterile services unit Round 1 (Q26–30). Sterile services unit Round 2 (Q1). Patient safety, quality and experience Round 1 (Q1–22, Q30–31). Patient safety, quality and experience Round 2 (Q1–6). Finance Round 1 (Q1–5, Q7–14, Q16–21, Q23, Q25–30). Finance Round 2 (Q1–6). Procurement Round 1 (Q1, Q17–19, Q23). Marketing & communications Round 1 (Q1–3, Q7–9, Q14, Q16, Q18–20, Q24–25, Q27). Medical risk-management/medicolegal Round 1 (Q1–23, Q28). Infrastructure Round 2 (Q3,6).
Domain 7: Vendor & Industry Support; Vendor Performance & Transparency	Evaluates whether industry partners provide adequate role specific training, education, guidance, and responsive support to enable safe adoption and sustained robotic practice, including transparency in service performance and risk disclosure.	Vendor responsibility; role specific education and training; early implementation support; simulation; service responsiveness; supply chain resilience; transparency	Anesthesia Round 1 (Q22). Perioperative nurses Round 1 (Q34–38). Biomedical engineers Round 1 (Q21–25). Biomedical engineers Round 2 (Q4–5, Q15). Sterile services unit Round 1 (Q21–24). Sterile services unit Round 2 (Q6–7). Finance Round 1 (Q22). Procurement Round 1 (Q11–12, Q20–22). Marketing & communications Round 1 (Q28). Medical risk-management/medicolegal Round 1 (Q24–27). Infrastructure Round 2 (Q5).
Domain 8: Operating Room Setup, Layout & Environmental Fit	Assesses whether robotic systems can be physically integrated into operating room environments without introducing hazards, congestion, or workflow inefficiencies.	Room layout; environmental safety; spatial efficiency; alert visibility	Biomedical engineers Round 2 (Q10–11). Perioperative nurses Round 1 (Q17–21). Perioperative nurses Round 2 (Q4). Infrastructure Round 1 (Q1–30). Infrastructure Round 2 (Q1–2, Q4). Procurement Round 1 (Q7).
Domain 9: Supplies, Logistics & Turnover	Focuses on the logistical demands of robotic surgery, ensuring that consumables, storage, equipment availability, and turnover processes support safe and efficient perioperative operations.	Supply resilience; turnover efficiency; equipment access	Biomedical engineers Round 1 (Q10–16). Biomedical engineers Round 2 (Q2). Perioperative nurses Round 1 (Q24–26). Procurement Round 1 (Q2–6, Q9, Q16). Sterile services unit Round 2 (Q4–5).

**Table 2:** Comparative overview of the three domain-weighted models. The table illustrates differences in domain prioritization across the clinician-weighted, patient safety-weighted, and procurement-weighted models. Weightings were developed using a normative, literature-informed approach to reflect distinct organizational perspectives, responsibilities, and decision-making priorities. Collectively, the models demonstrate how a common nine-domain framework can be applied through clinical, patient safety, and enterprise stewardship lenses while preserving a consistent evaluative structure.

Aspect	Clinician-Weighted Model	Safety-Weighted Model	Procurement-Weighted Model
Primary lens	Frontline clinical practice	Institutional patient safety and risk	Enterprise governance and capital management
Underlying mindset	Performance and usability focused	Harm avoidance and crisis containment	Sustainability, value preservation, and risk transfer
Dominant question	“Does this system let me operate and teach effectively every day?”	“Does this system introduce unacceptable risk?”	“Can this system be safely sustained and governed long-term?”
Highest priority domains	Technical capability, workflow, training	Patient safety, emergency preparedness, governance	Strategic governance, vendor performance, supply chain resilience
Role of patient safety	Essential but experiential and situational	Central and nonnegotiable	Baseline gating requirement
View of vendors and supply chains	Secondary unless failures disrupt cases	Important as contributors to safety risk	Central sources of financial and operational risk
Primary risk of overreliance	Underestimating enterprise and lifecycle risk	Over-constraining innovation	Undervaluing clinician experience and adoption

**Table 3:** Comparative weighting of thematic domains across the three evaluation models. Domain weightings for the clinician-weighted, patient safety-weighted, and procurement-weighted models. Weightings reflect differing organizational priorities and accountability perspectives relevant to robotic surgery evaluation, adoption, implementation, and governance. Detailed justification for the assigned weightings is provided in Supplementary Section S2.

Thematic Domain	Clinician-Weighted Model (%)	Safety-Weighted Model (%)	Procurement-Weighted Model (%)
Domain 1: Patient Safety & Risk Management; Emergency Preparedness & Crisis Management	18	22	18
Domain 2: Technical Capability & Surgical Performance	20	12	9
Domain 3: Intraoperative Workflow & Operational Integration	15	10	7
Domain 4: Training, Skill Acquisition & Trainee Governance	14	8	4
Domain 5: Credentialing, Supervision & Educational Infrastructure	12	8	4
Domain 6: Evaluation, Strategic Governance, Procurement, Adoption & Sustainability	9	20	25
Domain 7: Vendor & Industry Support; Vendor Performance & Transparency	2	8	15
Domain 8: OR Setup, Layout & Environmental Fit	6	6	8
Domain 9: Supplies, Logistics & Turnover	4	6	10
Total	100	100	100

**Table 4:** Categorization of consensus-derived items within the robotic system adoption framework. Consensus-derived items were organized into three complementary assessment categories: Institutional Readiness Assessment, Comparative Platform Evaluation, and Governance and Implementation Requirements. This categorization was designed to support structured evaluation and decision-making across the robotic surgery lifecycle, from pre-procurement readiness assessment and platform selection to implementation, oversight, and ongoing program governance. The complete 417-item instrument is available from the corresponding author upon reasonable request.

Thematic Category	Definition	Key Focus Areas	Included Question Numbers
Category 1: Institutional Readiness for Robotic Surgery Implementation	Assesses the extent to which a hospital possesses the organizational, workforce, governance, training, and workflow capacity required to safely adopt and sustain robotic surgery, independent of any specific robotic platform.	Organizational preparedness; workforce readiness; training infrastructure; credentialing and supervision; workflow integration; governance and outcomes monitoring	Surgeons Round 1 (Q26, Q38). Surgeons Round 2 (Q1, Q22). Perioperative nurses Round 1 (Q39). Biomedical engineers Round 1 (Q14). Biomedical engineers Round 2 (Q10–11). Sterile services unit Round 1 (Q6). Sterile services unit Round 2 (Q2–3). Finance Round 1 (Q4, Q6, Q28). Finance Round 2 (Q4). Infrastructure Round 1 (Q16–22, Q27–29).
Category 2: Comparative Evaluation of Surgical Robotic Systems	Supports hospital management and decisionmakers in evaluating and selecting among different surgical robotic platforms by focusing on system safety features, technical performance, usability, workflow efficiency, and functional capabilities aligned with clinical needs.	System safety and reliability; technical performance; visualization and dexterity; responsiveness; workflow efficiency; system versatility; educational and mentoring features	Surgeons Round 1 (Q1–2, 4, 6–15, Q17–18, Q30, Q33–37, Q39). Surgeons Round 2 (Q2–6, Q9, Q14–15, Q19–21). Anesthesiologists Round 1 (Q1–12, Q14–18, Q20, Q22–26). Anesthesiologists Round 2 (Q1–3). Perioperative nurses Round 1 (Q1–21, Q24–29, Q32–34, Q36–37). Perioperative nurses Round 2 (Q1–5). Biomedical engineers Round 1 (Q1–13, Q15–28). Biomedical engineers Round 2 (Q1–4, Q6–8, Q12, Q14–18). Sterile services unit Round 1 (Q1–5, Q7, Q9–14, Q16–17, Q19, Q21–24, Q28–30). Sterile services unit Round 2 (Q1, Q4–7). Patient safety, quality and experience Round 1 (Q7, Q9, Q19, Q23–24, Q27). Finance Round 1 (Q1–3, Q5, Q7–14, Q16–23, Q25–27). Finance Round 2 (Q1–2, Q6). Procurement Round 1 (Q1–14, Q16–22). Marketing & communications Round 1 (Q28). Medical risk management/medicolegal Round 1 (Q3, Q24, Q26–27). Infrastructure Round 1 (Q5, Q10–13). Infrastructure Round 2 (Q5).
Category 3: Prescriptive Institutional Requirements for Robotic Surgery Programs	Defines normative expectations, policies, and governance principles that institutions should establish when implementing robotic surgery, regardless of implementation timing or specific system choice.	Safety governance; training and credentialing standards; proctorship and oversight; procurement principles; workforce assessment; continuous performance monitoring	Surgeons Round 1 (Q3, Q5, Q16, Q19–25, Q27–29, Q31–32, Q40). Surgeons Round 2 (Q7–8, Q10–13, Q16–18, Q23, Q25–31, Q33–43, Q45). Anesthesiologists Round 1 (Q13, Q19, Q21, Q27, Q29–33). Perioperative nurses Round 1 (Q30–31, Q35, Q38, Q40). Perioperative nurses Round 2 (Q6). Biomedical engineers Round 1 (Q29–30). Biomedical engineers Round 2 (Q13, Q19–21). Sterile services unit Round 1 (Q8, Q26–27). Patient safety, quality and experience Round 1 (Q1–6, Q8, Q10–18, Q20–22, Q25–26, Q28, Q30–31). Patient safety, quality and experience Round 2 (Q1–6). Finance Round 1 (Q29–30). Finance Round 2 (Q3, Q5). Marketing & communications Round 1 (Q1–Q3, Q7–9, Q14, Q16, Q18–20, Q24–25, Q27). Medical risk management/medicolegal Round 1 (Q1–2, Q4–23, Q28–30). Infrastructure Round 1 (Q1–4, Q6–9, Q14–15, Q23–26, Q30). Infrastructure Round 2 (Q1–4, Q6). Procurement Round 1 (Q15, Q23).

**Table 5:** Institutional Readiness Assessment composite score interpretation rubric. Interpretation framework for normalized domain-weighted scores (0–100) used to evaluate organizational readiness for the adoption, implementation, and long-term sustainability of robotic surgery programs. The rubric provides guidance for interpreting institutional strengths, capability gaps, and readiness for program deployment.

Composite Score Range (%)	Interpretation	Recommended Action
≥80	Strong Alignment	The system or institutional readiness is highly aligned with the selected priority model, with no major structural gaps identified. Leadership may proceed to the next decision phase (e.g., contract finalization or pilot deployment) while continuing to monitor lower-scoring domains.
65–79	Conditional Alignment	Overall alignment is acceptable, but important weaknesses exist in one or more domains relative to institutional priorities. Progression may be considered only with targeted mitigation plans addressing identified gaps, such as training, safety workflows, governance, or vendor support.
50–64	Marginal Alignment	Multiple priority domains demonstrate inadequate performance under the selected model, creating elevated organizational, operational, or patient safety risks. Adoption should be deferred pending targeted remediation, capability development, or vendor renegotiation.
<50	Poor Alignment	The system or institutional readiness is fundamentally misaligned with institutional priorities under the selected model. Leadership should not proceed and should instead reassess strategic objectives, alternative technologies, or readiness assumptions before reconsideration.

**Table 6:** Comparative Platform Evaluation composite score interpretation rubric. Interpretation framework for normalized domain-weighted scores (0–100) used to assess and compare the relative performance, suitability, and strategic fit of competing robotic surgery platforms. The rubric supports structured comparison across clinical, operational, technical, governance, and organizational domains.

Composite Score Range (%)	Interpretation	Recommended Action
≥85	Highly Favorable Platform	Strong candidate for procurement
75–84	Favorable Platform	Suitable for procurement with minor limitations
65–74	Acceptable Platform	Procurement conditional upon local priorities
50–64	Marginal Platform	Significant limitations identified
<50	Unfavorable Platform	Do not proceed unless major justification exists

**Table 7:** Governance and Implementation Maturity Assessment composite score interpretation rubric. Interpretation framework for normalized domain-weighted scores (0–100) used to evaluate the maturity of governance structures, implementation processes, oversight mechanisms, and quality assurance systems supporting robotic surgery programs. The rubric supports assessment of organizational capability to sustain safe, effective, and accountable program operations over time.

Composite Score Range (%)	Interpretation	Recommended Action
≥85	Optimized Governance	Mature robotic program
75–84	Managed Governance	Governance framework well established
65–74	Defined Governance	Acceptable but improvement needed
50–64	Emerging Governance	Significant governance gaps
<50	Immature Governance	Governance structure inadequate

**Table 8:** Stakeholder prioritization of domains in robotic surgery adoption. Each column represents the rankings assigned by a stakeholder group to the thematic domains listed in the rows. Domains were ranked according to perceived importance on a scale from 1 (most important) to 9 (least important). Rankings assigned by all stakeholder groups were summed to generate a total score for each domain. Lower total scores indicate higher overall perceived importance across stakeholder groups and reflect areas considered most influential in robotic surgery adoption, implementation, and governance.

Domain	Anesthesiologists	Finance Leaders	Surgeons	Patient Safety, Quality and Experience Leaders	Procurement Leaders	Medical Risk Management/Medicolegal Leaders	Infrastructure Leaders	Perioperative Nurses	Sterile Services Staff	Biomedical Engineer Leaders	Total Score (Sum of all the Columns)
Domain 1: Patient Safety & Risk Management; Emergency Preparedness & Crisis Management	1	1	1	6	2	1	1	1	1	2	17
Domain 2: Technical Capability & Surgical Performance	2	3	2	1	3	3	4	3	2	1	23
Domain 3: Intraoperative Workflow & Operational Integration	3	5	3	2	7	2	3	2	5	5	37
Domain 4: Training, Skill Acquisition & Trainee Governance	4	6	4	3	8	4	9	4	4	4	50
Domain 6: Evaluation, Strategic Governance, Procurement, Adoption & Sustainability	6	2	6	4	1	6	5	9	9	7	55
Domain 5: Credentialing, Supervision & Educational Infrastructure	5	9	5	7	9	8	6	5	3	3	60
Domain 8: Operating Room Setup, Layout & Environmental Fit	8	8	8	5	4	9	2	8	6	8	66
Domain 7: Vendor & Industry Support; Vendor Performance & Transparency	7	4	7	8	5	5	7	7	7	6	73
Domain 9: Supplies, Logistics & Turnover	9	7	9	9	6	7	8	6	8	9	78

**Table 9:** Stakeholder prioritization of stakeholder group influence in robotic surgery adoption. Each column represents the rankings assigned by a stakeholder group to all stakeholder groups listed in the rows. Stakeholder groups were ranked according to their perceived influence on robotic surgery adoption decisions, with 1 indicating the greatest influence and 12 indicating the least influence. Rankings assigned by all stakeholder groups were summed to generate a total score for each stakeholder group. Lower total scores indicate greater perceived influence within institutional decision-making related to robotic surgery evaluation, adoption, implementation, and governance.

Stakeholder Group	Anesthesiologists	Finance Leaders	Surgeons	Patient Safety, Quality and Experience Leaders	Procurement Leaders	Medical Risk Management/Medicolegal Leaders	Infrastructure Leaders	Perioperative Nurses	Sterile Services Staff	Biomedical Engineer Leaders	Total Score (Sum of all the Columns)
Surgeons	1	2	1	2	2	3	2	2	1	1	17
Patient Safety, Quality and Experience	5	1	8	1	1	4	1	7	4	11	43
Perioperative Nurses	3	7	9	3	4	7	4	1	2	4	44
Anesthesiologists	2	6	6	5	3	8	5	3	3	5	46
Medical Risk Management & Medicolegal	9	3	3	6	8	2	3	8	8	3	53
Biomedical Engineers	4	8	2	9	5	6	6	5	6	7	58
Infrastructure	8	5	10	4	9	1	7	6	7	8	65
Sterile Supply Unit	6	10	5	11	11	5	8	4	5	6	71
Finance	7	4	4	7	10	9	9	10	10	9	79
CEO/Senior Leadership	10	9	7	10	7	11	10	11	11	2	88
Procurement	11	11	11	8	6	10	11	9	9	10	96
Marketing and Communications	12	12	12	12	12	12	12	12	12	12	120

## Data Availability

The datasets used and analyzed during the current study are available from the corresponding author on reasonable request. As the data is currently being included in another larger study, the authors are refraining from putting the data on public domain for now.

## Supplementary Materials

The following supporting information can be downloaded at: https://www.mdpi.com/article/10.3390/healthcare14162637/s1, Section S1: Composition of Stakeholder Groups. Section S2: Rationale for Domain-Weighted Models. Section S3: Participant Information Sheet and Informed Consent Form. Section S4: Conflict of Interest Self-Declarations. Section S5: Worked Example for Hypothetical Hospitals A (Clinically-Focused Hospital) vs. Hospital B (Enterprise-Focused Hospital) and How to Apply the Scoring Rubric. Section S6: Delphi-Based Refinement of Item Wording. Section S7. Sensitivity Analysis of the Weighting Framework.
