# Supplementary material for: From Evaluation to Implementation: A Delphi Consensus Framework with Domain-Weighted Decision Models for Robotic Surgery Adoption
Source: Healthcare (Basel). 2026 Aug 20;14(16):2637. doi: 10.3390/healthcare14162637 (PMC13512483; doi:10.3390/healthcare14162637)
Supplement: Supplementary file 1 [file healthcare-14-02637-s001.zip › healthcare-4453031-supplementary.pdf]

Supplementary Section S1: Composition of Stakeholder Groups

For the purposes of this study, participants were categorized into two broad groups: surgical stakeholders and non-surgical stakeholders.

Within the surgical stakeholder group, senior surgeons were defined as clinicians with ⩾5 years of independent robotic surgical experience, while junior surgeons were defined as those with <5 years of independent robotic surgical experience.

Within the non-surgical stakeholder group, senior staff were defined as individuals with ⩾5 years of experience supporting the implementation, operation, or governance of robotic surgery programs, whereas junior staff were defined as those with <5 years of such experience.

A detailed breakdown of stakeholder participation is provided in Table S1, including the composition of each stakeholder group, the number of participants, and the distribution of respondents by seniority level. Seniority was determined according to years of experience in robotic surgery practice or in supporting robotic surgery implementation and operations.

Table S1. Characteristics of Study Participants by Stakeholder Group and Seniority

Stakeholder Group	Total Participants (n)	Senior Staff (⩾5 Years' Experience) (n)	Junior Staff (<5 Years' Experience) (n)	Senior Staff (%)	Junior Staff (%)
**Surgeons**	9	5	4	57	43
**Patient Safety, Quality and Experience**	18	12	6	67	33
**Perioperative Nurses**	32	21	11	66	34
**Anesthesiologists**	12	8	4	67	33
**Medical Risk Management & Medicolegal**	7	4	3	57	43
**Biomedical Engineers**	19	13	6	68	32
**Infrastructure**	19	12	7	63	37
**Sterile Supply Unit**	17	11	6	65	35

**Finance**	8	4	4	50	50
**Procurement**	17	10	7	59	41
**Marketing and Communications**	15	8	7	53	47

Several stakeholder groups, including Finance (n = 8) and Medical Risk Management & Medicolegal (n = 7), had relatively small sample sizes. Consequently, consensus estimates within these groups should be interpreted with caution, as agreement proportions are inherently more sensitive to the views of individual participants. For example, in smaller stakeholder groups, a change in the response of a single participant may result in a meaningful shift in the calculated consensus proportion. However, the objective of this study was not to generate statistically representative estimates for individual departments, but rather to obtain multidisciplinary input from stakeholders involved in the adoption, implementation, governance, and operational support of robotic-assisted surgery. To enhance the credibility of the findings, stakeholder groups were identified *a priori* based on their expected roles in robotic surgery programs, and both senior and junior staff were represented across all groups. Furthermore, the final weighting framework was informed by convergence of views across multiple stakeholder categories rather than being driven by any single group's responses. Future studies with larger sample sizes, particularly within smaller stakeholder groups, would be valuable to further examine the stability and generalizability of the consensus findings.

Supplementary Section S2: Rationale for Domain-Weighted Models

The three domain-weighted models were developed to reflect different organizational perspectives commonly encountered during robotic surgery evaluation, procurement, implementation, and governance. Rather than representing statistically derived weightings, the models make explicit the relative importance assigned to each domain under different accountability and decision-making lenses.

The assigned weightings reflect normative judgments regarding the extent to which deficiencies within a domain may affect clinical effectiveness, patient safety, organizational performance, or long-term sustainability. Their purpose is not to identify a single optimal weighting scheme, but to illustrate how priorities may legitimately differ according to stakeholder responsibilities and institutional objectives.

Clinician-Weighted Model

The clinician-weighted model assigns its highest weighting to Technical Capability & Surgical Performance (20%), reflecting the central role of this domain in frontline surgical practice. From a clinician's perspective, robotic systems are primarily operative tools. Factors such as instrument dexterity, visualization quality, system responsiveness, ergonomics, and overall usability directly influence procedural efficiency, learning curves, operator fatigue, confidence, and potentially patient outcomes.

Patient Safety & Risk Management; Emergency Preparedness & Crisis Management (18%) receives the second-highest weighting. Clinicians experience safety directly through patient care and must respond in real time to intraoperative complications, including bleeding, airway emergencies, and system failures. Consequently, safe system design and effective integration into operative workflows are essential for timely recognition, escalation, and management of adverse events. The slightly lower weighting relative to technical performance reflects the close relationship between usability and safety in routine clinical practice.

Substantial weight is also allocated to Intraoperative Workflow & Operational Integration (15%), Training, Skill Acquisition & Trainee Governance (14%), and Credentialing, Supervision & Educational Infrastructure (12%). Together, these domains account for over half of the total weighting and reflect clinician concerns regarding operational efficiency, cognitive workload, competency development, and professional accountability. These factors collectively shape confidence in a robotic system's ability to support safe and effective surgical practice.

By contrast, Evaluation, Strategic Governance, Procurement, Adoption & Sustainability (9%), OR Setup, Layout & Environmental Fit (6%), Supplies, Logistics & Turnover (4%), and Vendor & Industry Support; Vendor Performance & Transparency (2%) receive lower weightings. Although important, these domains are generally less visible to frontline clinicians unless deficiencies directly affect clinical operations or patient care.

Patient Safety-Weighted Model

The patient safety-weighted model assigns the greatest emphasis to Patient Safety & Risk Management; Emergency Preparedness & Crisis Management (22%), reflecting the principle that preventable patient harm represents a critical institutional failure. From a safety perspective, risks to patients cannot be offset by gains in efficiency, technical performance, or operational convenience, particularly when adverse events carry clinical, regulatory, legal, or reputational consequences.

A similarly high weighting is assigned to Evaluation, Strategic Governance, Procurement, Adoption & Sustainability (20%), recognizing that safety is not solely an intraoperative concern but a system-level property influenced by governance structures, accountability mechanisms, escalation pathways, monitoring processes, and lifecycle oversight. Weak governance may function as a latent safety threat that increases organizational vulnerability over time.

Technical Capability & Surgical Performance (12%) and Intraoperative Workflow & Operational Integration (10%) receive moderate weightings because of their contribution to error prevention, situational awareness, and safe procedural execution. Training, Skill Acquisition & Trainee Governance and Credentialing, Supervision & Educational Infrastructure (both 8%) are weighted primarily as mechanisms for reducing risk and maintaining competency.

Lower but still meaningful weightings are assigned to vendor performance, infrastructure, and logistics domains. These factors remain relevant to patient safety but are generally considered manageable through effective governance, contractual safeguards, organizational controls, and proactive monitoring.

Procurement-Weighted Model

The procurement-weighted model assigns its highest weighting to Evaluation, Strategic Governance, Procurement, Adoption & Sustainability (25%), reflecting the view that long-term value is determined not only by the technology itself but also by the institution's ability to govern, implement, and sustain its use effectively. Failures in governance, adoption planning, or lifecycle management may ultimately be more consequential than limitations in technical performance.

Vendor & Industry Support; Vendor Performance & Transparency (15%) receives the second-highest weighting. Procurement leaders are often responsible for managing risks associated with vendor dependence, service reliability, cybersecurity obligations, contractual flexibility, upgrade pathways, and long-term support arrangements. The substantially higher weighting assigned to this domain, compared with the clinician model, reflects the importance of vendor-related considerations in enterprise-level decision-making.

Patient Safety & Risk Management; Emergency Preparedness & Crisis Management (18%) remains highly weighted but functions primarily as a gating requirement. In practice, minimum safety standards must first be satisfied before meaningful differentiation can occur across other domains. Once safety requirements have been met, decision-making increasingly focuses on sustainability, financial exposure, operational resilience, and long-term organizational risk.

The increased weighting assigned to Supplies, Logistics & Turnover (10%) reflects recognition of supply-chain risks and the operational and financial consequences of equipment downtime, consumable shortages, service disruptions, or case cancellations.

Conversely, lower weightings are assigned to training, credentialing, and workflow-related domains because these issues are generally considered more amenable to institutional investment, change management, and governance processes than intrinsic characteristics of the robotic platform itself.

Institutional Choice and Strategic Alignment

The three weighting models should not be interpreted as competing alternatives or as representing a hierarchy of priorities. Rather, they provide complementary perspectives through which robotic surgery programs may be evaluated.

The relative importance assigned to each perspective is likely to vary according to factors such as organizational maturity, case mix, resource availability, governance structures, regulatory requirements, financial constraints, and strategic objectives.

For example, academic referral centers undergoing early adoption or program expansion may place greater emphasis on clinician-weighted considerations to support technical innovation, education, and procedural excellence. In contrast, smaller or resource-constrained institutions may prioritize procurement-weighted considerations to strengthen affordability, operational resilience, and protection against vendor lock-in. Organizations operating under heightened regulatory scrutiny or responding to significant safety events may choose to emphasize patient safety-weighted considerations to reinforce risk management and stakeholder confidence.

The explicit selection of a weighting model, or the parallel application of multiple models, enables institutions to align decision-making with strategic objectives while making underlying priorities and trade-offs transparent. Such an approach supports more structured, consistent, and defensible evaluations by clarifying both the relative importance and practical implications of different organizational priorities.

Importantly, these weighting models are intended as illustrative decision-support tools rather than prescriptive or universally applicable standards. Institutions may therefore adapt domain weightings to reflect their own governance arrangements, operational realities, strategic goals, and local context.

Supplementary Section S3: Participant Information Sheet and Informed Consent Form

Study Title: Development of a Multi-Stakeholder Consensus Framework for the Assessment and Implementation of Surgical Robotics in Southeast Asian Hospitals: A Delphi Study

Principal Investigators:

Dr. Kenneth Chen, MBBS, MMed (Surgery), FRCS — SingHealth, Singapore

Poon Tsi Yu, MSc — SingHealth, Singapore

1. Introduction

You are invited to participate in a research study exploring the assessment, adoption, and implementation of surgical robotic platforms in hospitals across Southeast Asia. You have been approached because you are recognized as a key opinion leader or domain expert in your field. Your insights will help shape a regional, multi-stakeholder framework intended to guide hospital leaders and policymakers in making evidence-informed decisions about surgical robotics. Please read the following information carefully. You may ask any questions before deciding whether to participate. Participation is entirely voluntary.

2. Purpose of the Study

The purpose of this study is to develop a consensus-based, leadership-ready framework for evaluating and implementing surgical robotic systems in Southeast Asian hospitals. The study uses a multi-round Delphi process involving 11 stakeholder groups, including clinicians, nursing teams, sterile services staff, biomedical engineers, administrators, finance, procurement, legal, cybersecurity, and patient safety leaders. Your contributions will help identify key considerations, readiness criteria, and implementation indicators relevant to your area of expertise.

3. Study Procedures

If you agree to participate, you will be asked to complete:

Round 1: A role-specific questionnaire containing Likert items and open-ended questions. Estimated time: 15–25 minutes.

Round 2: A refined questionnaire presenting the aggregated responses from Round 1. You will be asked to re-rate items and comment on revised or newly added indicators. Estimated time: 10–20 minutes.

All participation will be conducted online via a secure survey platform. You may skip any question or stop participation at any time.

4. Potential Risks and Discomforts

This study involves minimal risk. Possible inconveniences may include: minor time commitment to complete the surveys, and mild fatigue from reading or rating items. There are no known physical, psychological, legal, or financial risks associated with participation.

5. Potential Benefits

You may not receive direct personal benefit. However, your participation will contribute to: (1) A regionally relevant framework for surgical robotics adoption, (2) Improved decision-making processes in hospitals across Southeast Asia, and (3) Enhanced safety, value, and

sustainability of surgical robotics programs. You may also gain professional insight into regional practices and consensus viewpoints.

6. Voluntary Participation and Right to Withdraw

Participation is entirely voluntary. You may decline to participate, skip any question, and withdraw from the study at any time without penalty or explanation. Your decision will not affect your professional standing or relationship with any institution.

7. Confidentiality and Data Protection

Your identity will be kept strictly confidential. No personally identifiable information will be collected in the survey response forms. All responses will be anonymized and stored securely on encrypted servers accessible only to the study investigators. Data will be reported only in aggregated form, with no way to trace responses back to any individual. If quotations from your open-ended responses are used in publications, they will be fully de-identified. Data will be retained securely for the period required by institutional policies.

8. Use of Artificial Intelligence Tools

Artificial intelligence (AI) language models were used only in the initial drafting of survey items. All survey content underwent expert human review and revision. No AI tools will be used to process personal or identifiable information.

9. Compensation

You will not receive any financial compensation for participation. There are no costs to you.

10. Funding Disclosure

This study is conducted without external funding. No grants, sponsorship, or commercial support were received.

11. Conflicts of Interest

The investigators declare no conflicts of interest related to this study or the use of surgical robotic technologies.

12. Ethics Approval

This study protocol has been reviewed and approved by the coordinating institution's Institutional Review Board (IRB). Additional ethics approvals or waivers were obtained in participating countries as required by local regulations. The study complies with the Declaration of Helsinki and all applicable ethical guidelines.

13. Contact Information

If you have questions about the study, please contact:

Principal Investigator:

Dr. Kenneth Chen, MBBS, MCI, MRCS (Edin), FRCS (Urol, Glasg), FAMS (Urol), Senior Consultant Surgeon, SGH Department of Urological Surgery, Singapore.

Email: kenneth.chen@singhealth.com.sg

Co-Investigator:

Poon Tsi Yu, MSc

SingHealth, Singapore.

Email: poon.tsi.yu@singhealth.com.sg

If you have questions about your rights as a research participant, or wish to contact an independent party not involved in the study, you may

contact the SingHealth Centralized Institutional Review Board (CIRB) at +65 8126 3660 during office hours, or by email at irb@singhealth.com.sg. The CIRB office is located at 20 College Road, Academia Level 6, Singapore 169856.

14. Consent Statement

By clicking "I agree" or proceeding to complete the questionnaire, you confirm that:

You have read and understood the information provided;

Your questions have been answered;

You voluntarily agree to participate in this study.

Supplementary Section S4: Conflict of Interest Self-Declarations

Author Conflict of Interest Declaration

The author(s) declare no actual, potential, or perceived conflicts of interest. Specifically, the author(s) have no financial relationships, advisory roles, equity interests, or other affiliations with manufacturers or vendors of surgical robotic systems that could influence the conduct or outcomes of this study. Participants in the Delphi process were hospital stakeholders involved in the assessment and procurement of surgical robotic technologies and contributed their views solely in a professional and expert capacity. Measures were taken to ensure anonymity of responses and to mitigate potential institutional or commercial bias. No external commercial funding was received for this study, and no commercial entity had any role in the study design, data analysis, interpretation of results, or manuscript preparation.

Participant Conflict of Interest Declaration

By participating in this Delphi consensus study, I declare that my contributions are provided in my professional capacity. I have disclosed any current or recent (within the past three years) financial, professional, or other relationships that could be perceived as influencing my views on the assessment or procurement of surgical robotic systems, including but not limited to relationships with manufacturers, vendors, distributors, or commercial affiliates of surgical robotic technologies. I understand that participation in this study is voluntary, that responses will be anonymized, and that disclosed conflicts of interest will be considered in the interpretation and reporting of study findings.

Supplementary Section S5: Worked Example for Hypothetical Hospitals A (Clinically-Focused Hospital) vs. Hospital B (Enterprise-Focused Hospital) and How to Apply the Scoring Rubric.

Worked Example: Hypothetical Hospital Profiles

To illustrate the practical application of the framework, two hypothetical hospitals with differing organizational priorities and maturity profiles were assessed using the domain-weighted models.

Hospital A represents a clinically driven academic medical center focused on surgical excellence, innovation, and workforce development. The institution demonstrates strong surgeon engagement, robust leadership support for robotic surgery, and well-established training, credentialing, and educational processes. Multidisciplinary workflow planning has been extensively undertaken, and operating room teams are experienced in managing complex surgical technologies. However, governance processes remain moderately developed, with gaps in long-term program oversight, vendor management, contract governance, and lifecycle planning. Supply-chain resilience and vendor performance management are still evolving. Consequently, Hospital A would be expected to perform favorably under the clinician-weighted model, which prioritizes technical capability, workflow integration, training, and clinical operations.

Hospital B represents an enterprise-oriented healthcare organization that views robotic surgery primarily through the lens of strategic investment and organizational sustainability. The institution has mature governance structures, strong financial planning processes, comprehensive procurement oversight, and well-developed vendor management capabilities. Considerable attention has been devoted to infrastructure readiness, lifecycle cost assessment, supply-chain resilience, and long-term program sustainability. However, its robotic surgery program is relatively new, resulting in less mature training pathways, lower workforce experience, and less developed workflow integration compared with Hospital A. Accordingly, Hospital B would be expected to perform most strongly under the procurement-weighted model, which emphasizes governance, sustainability, vendor performance, financial stewardship, and long-term organizational value.

These contrasting profiles illustrate how the same framework may produce different assessments when applied through different institutional perspectives.

Framework Application and Composite Scoring Methodology

The 417-item instrument is organized into nine thematic domains and three analytical categories:

1. Institutional Readiness Assessment (Category 1)

Assesses the extent to which a hospital possesses the organizational, workforce, infrastructure, governance, operational, and patient safety capabilities necessary to establish and sustain a robotic surgery program.

2. Comparative Robotic Platform Evaluation (Category 2)

Supports comparison of competing robotic platforms based on technical performance, safety features, functionality, usability, workflow implications, and platform-specific capabilities.

3. Governance and Implementation Requirements (Category 3)

Provides implementation guidance and governance recommendations required to support safe, effective, and sustainable robotic surgery programs. These items are not included in composite readiness scoring.

For institutional readiness assessment, Category 1 items are scored using the nine-point Likert scale employed throughout the study. Item-level scores are aggregated within each domain and normalized to generate a domain score ranging from 0% to 100%.

Table S2: Comparative Scores for Hypothetical Hospitals A and B Across Nine Assessment Domains

Domain	Hospital A	Hospital B
Domain 1: Patient Safety & Risk Management; Emergency Preparedness & Crisis Management	85	82
Domain 2: Technical Capability & Surgical Performance	95	75
Domain 3: Intraoperative Workflow & Operational Integration	90	70
Domain 4: Training, Skill Acquisition & Trainee Governance	92	60
Domain 5: Credentialing, Supervision & Educational Infrastructure	88	65
Domain 6: Evaluation, Strategic Governance, Procurement, Adoption & Sustainability	70	95
Domain 7: Vendor & Industry Support; Vendor Performance & Transparency	60	92
Domain 8: OR Setup, Layout & Environmental Fit	75	90
Domain 9: Supplies, Logistics & Turnover	65	90

Following calculation of domain scores, institutions apply the domain-weighted model that best aligns with their strategic priorities. Domain scores are multiplied by the corresponding weighting factors and summed to generate a composite readiness score (0-100).

Hospital A: Clinician-Weighted Model

Domain	Score	Weight	Contribution
Domain 1: Patient Safety & Risk Management; Emergency Preparedness & Crisis Management	85	18%	15.3
Domain 2: Technical Capability & Surgical Performance	95	20%	19.0
Domain 3: Intraoperative Workflow & Operational Integration	90	15%	13.5

Domain 4: Training, Skill Acquisition & Trainee Governance	92	14%	12.9
Domain 5: Credentialing, Supervision & Educational Infrastructure	88	12%	10.6
Domain 6: Evaluation, Strategic Governance, Procurement, Adoption & Sustainability	70	9%	6.3
Domain 7: Vendor & Industry Support; Vendor Performance & Transparency	60	2%	1.2
Domain 8: OR Setup, Layout & Environmental Fit	75	6%	4.5
Domain 9: Supplies, Logistics & Turnover	65	4%	2.6

Composite Score = 85.9 / 100

Interpretation: Strong Alignment (Table 5; ≥80)

From a clinical implementation perspective, Hospital A demonstrates a high degree of readiness for robotic surgery adoption. Strong performance in technical capability, workflow integration, training infrastructure, and credentialing compensates for moderate weaknesses in governance maturity, vendor performance oversight, and supply-chain resilience. Under this evaluative lens, the institution would be considered ready to proceed with implementation, subject to targeted mitigation strategies focused on governance and vendor management.

Hospital B: Procurement-Weighted Model

Domain	Score	Weight	Contribution
Domain 1: Patient Safety & Risk Management; Emergency Preparedness & Crisis Management	82	18%	14.8
Domain 2: Technical Capability & Surgical Performance	75	9%	6.8
Domain 3: Intraoperative Workflow & Operational Integration	70	7%	4.9
Domain 4: Training, Skill Acquisition & Trainee Governance	60	4%	2.4
Domain 5: Credentialing, Supervision & Educational Infrastructure	65	4%	2.6
Domain 6: Evaluation, Strategic Governance, Procurement, Adoption & Sustainability	95	25%	23.8
Domain 7: Vendor & Industry Support; Vendor Performance & Transparency	92	15%	13.8
Domain 8: OR Setup, Layout & Environmental Fit	90	8%	7.2
Domain 9: Supplies, Logistics & Turnover	90	10%	9.0

Composite Score = 85.3 / 100

Interpretation: Strong Alignment (Table 5; ≥80)

From an enterprise stewardship perspective, Hospital B demonstrates substantial organizational readiness. Governance structures, procurement controls, vendor management processes,

infrastructure planning, and supply-chain resilience are highly developed. Although training pathways and clinical workflow integration remain relatively immature, these are considered manageable organizational risks within the procurement-weighted model. Under this lens, the institution would be considered suitable to proceed with robotic surgery procurement and implementation, accompanied by targeted workforce development initiatives.

Illustrative Effect of Different Weighting Models

One of the key features of the framework is that the same institution may generate different readiness assessments depending on the evaluative lens applied.

Table S3. Comparison of Assessment Scores for Hypothetical Hospital A Under Clinician-Weighted and Procurement-Weighted Models

Model	Composite Score
Clinician-Weighted	85.9
Procurement-Weighted	76.2

Although Hospital A demonstrates strong readiness under the clinician-weighted model, its score decreases when assessed using the procurement-weighted model. This reduction is driven by lower performance in governance, vendor management, and supply-chain resilience, domains that carry greater importance within an enterprise stewardship framework. The example illustrates how an institution may appear highly prepared from a frontline clinical perspective while still exhibiting meaningful weaknesses from a governance and sustainability perspective.

This worked example demonstrates how the framework can be used not only to assess organizational readiness, but also to identify readiness gaps, guide remediation efforts, and support transparent, evidence-informed leadership decision-making regarding robotic surgery adoption.

Supplementary Section S6: Delphi-Based Refinement of Item Wording

Clarification of the refinement process

Consistent with established Delphi methodology, candidate items were iteratively refined to improve clarity, precision, and interpretability. Revisions included clarification of wording, refinement of definitions, neutralization of directive or prescriptive language, and restructuring of item phrasing. Where items encompassed multiple concepts, these were separated into individual statements when panelists identified them as distinct organizational processes with differing levels of perceived importance.

For example, items that combined governance and operational considerations within a single statement were divided into separate items to enable independent evaluation of each construct. Similarly, items containing potentially ambiguous terminology were revised using more specific language to enhance consistency of interpretation across stakeholder groups. Where appropriate, items were reframed to assess the perceived importance of a concept rather than to prescribe a particular action, practice, or organizational approach.

Three examples of questions that underwent Delphi refinement

Original Delphi Round 1 Questions & Responses

Question 1: "What training or curriculum elements should be prioritized to ensure safe and effective integration of robotic surgery into your department?"

Expert Opinion (Raw Response): Structured pre-operative training for surgeon assistants and nurses in a team.

Step 1: Thematic Categorization.

Identified Primary Theme: Team Based Pre Operative Training

This response emphasizes that safe robotic adoption depends on team readiness, not surgeon training alone.

Step 2: Theme and Subtheme Mapping.

**Theme**	**Subtheme**	**Interpretation**
Multidisciplinary Training	Surgeon assistants and nurses	Robotic safety is team dependent
Pre Operative Preparedness	Structured training before clinical use	Reduces intraoperative errors
Team Coordination	Shared understanding of system use	Improves workflow and response

———————————————————

Step 3: Refined Thematic Statement for Delphi Traceability: Safe integration of robotic surgery requires structured, preoperative training for the entire perioperative team, including surgeon assistants and nursing staff.

———————————————————

Step 4: Refined Likert Item for Round 2: Structured preoperative training for surgeons, assistants, and nursing staff supports the safe and effective integration of robotic surgery.

———————————————————

Question 2: "What challenges, risks, or underestimated barriers do you foresee in incorporating robotic surgery into surgical training programs?"

Expert Opinion (Raw Response): Not every specialty will find robotic surgery applicable, and different specialties may require different types of robotic platforms.

———————————————————

Step 1: Thematic Categorization.

Identified Primary Theme: Specialty Specific Applicability & Platform Heterogeneity

This response highlights variation in clinical relevance and technology needs across specialties.

———————————————————

Step 2: Theme and Subtheme Mapping.

Theme	Subtheme	Interpretation
Clinical Relevance Variation	Not all specialties benefit	Training demand may differ
Technology Diversity	Different robotic platforms	Reduces standardization
Training Complexity	Platform specific learning	Increases cognitive burden

———————————————————

Step 3: Refined Thematic Statement for Delphi Traceability: Differences in specialty applicability and the need for multiple robotic platforms may complicate training program design and uptake.

Step 4: Refined Likert Item for Round 2: Variation in specialty applicability and robotic platform design poses challenges to standardized robotic surgery training programs.

Question 3: "What system features, supports, or non-negotiable requirements are essential to ensure safe, supervised adoption of robotic surgery?"

Expert Opinion (Raw Response): Safety features and fault management mechanisms that maintain patient safety during unexpected situations such as bleeding.

Step 1: Thematic Categorization.

Identified Primary Theme: Intraoperative Safety & Fault Tolerance

This response focuses on robust system behavior during emergencies.

Step 2: Theme and Subtheme Mapping.

Theme	Subtheme	Interpretation
System Safety Features	Built in safeguards	Prevents harm
Fault Management	Response to technical failure	Maintains control
Emergency Readiness	Unexpected bleeding scenarios	High risk moments

---

Step 3: Refined Thematic Statement for Delphi Traceability: Safe adoption of robotic surgery requires system level safety features and fault management mechanisms that protect patients during unexpected intraoperative events.

Step 4: Refined Likert Item for Round 2: Robotic systems should include robust safety and fault management mechanisms to maintain patient safety during unexpected intraoperative events.

Supplementary Section S7. Sensitivity Analysis of the Weighting Framework

Deterministic and Probabilistic Sensitivity Analysis

To assess the robustness of the weighted scoring framework, deterministic and probabilistic sensitivity analyses were conducted using hypothetical simulated domain performance profiles. The analyses assessed whether reasonable perturbations in domain weightings would materially alter composite scores or recommendation categories and were designed as illustrative tests of framework stability.

In the deterministic analysis, individual domain weights were perturbed by ±5 percentage points while maintaining a total weighting of 100%. Across all tested scenarios, composite scores varied by less than 3.5 points from baseline values. High-performing and poor-performing solutions retained their recommendation categories in every scenario. Only the borderline profile, which was positioned near the recommendation threshold, demonstrated limited category transitions under extreme perturbations.

A Monte Carlo simulation involving 10,000 random weight combinations per stakeholder model further demonstrated robustness. Domain weights were allowed to vary within ±5 percentage points and were subsequently normalized to maintain a total weighting of 100%. Across simulations, high-performing and poor-performing archetypes retained their recommendation categories in ⩾99% of iterations, while the borderline profile retained its category in approximately 93-96% of iterations. Composite score variation remained small, with standard deviations generally below 1.5 points.

Despite substantial variation in domain emphasis across clinician-, safety-, and procurement-weighted models, recommendation categories remained unchanged for clear high-performing and poor-performing solutions, indicating strong decision robustness. The findings suggest that assessment outcomes are stable and not materially sensitive to reasonable variations in stakeholder weighting preferences. As expected, sensitivity was greatest among solutions located near category boundaries, where small changes in weighting can influence classification outcomes.

Three stakeholder weighting models were evaluated:

- Clinician-weighted model
- Safety-weighted model
- Procurement-weighted model

Simulated Performance Profiles

Three hypothetical solutions representing different performance archetypes were modelled.

High-Performing Solution

Domain	Score
Domain 1: Patient Safety & Risk Management; Emergency Preparedness & Crisis Management	90
Domain 2: Technical Capability & Surgical Performance	92
Domain 3: Intraoperative Workflow & Operational Integration	88
Domain 4: Training, Skill Acquisition & Trainee Governance	87
Domain 5: Credentialing, Supervision & Educational Infrastructure	86
Domain 6: Evaluation, Strategic Governance, Procurement, Adoption & Sustainability	89
Domain 7: Vendor & Industry Support; Vendor Performance & Transparency	84
Domain 8: OR Setup, Layout & Environmental Fit	85
Domain 9: Supplies, Logistics & Turnover	86

Expected overall score: approximately 88-90

Borderline Solution

Domain	Score
Domain 1: Patient Safety & Risk Management; Emergency Preparedness & Crisis Management	72
Domain 2: Technical Capability & Surgical Performance	74
Domain 3: Intraoperative Workflow & Operational Integration	69
Domain 4: Training, Skill Acquisition & Trainee Governance	68
Domain 5: Credentialing, Supervision & Educational Infrastructure	67
Domain 6: Evaluation, Strategic Governance, Procurement, Adoption & Sustainability	71
Domain 7: Vendor & Industry Support; Vendor Performance & Transparency	64
Domain 8: OR Setup, Layout & Environmental Fit	69
Domain 9: Supplies, Logistics & Turnover	70

Expected overall score: approximately 69-71

Poor-Performing Solution

Domain	Score
Domain 1: Patient Safety & Risk Management; Emergency Preparedness & Crisis Management	58
Domain 2: Technical Capability & Surgical Performance	55
Domain 3: Intraoperative Workflow & Operational Integration	53
Domain 4: Training, Skill Acquisition & Trainee Governance	52
Domain 5: Credentialing, Supervision & Educational Infrastructure	50
Domain 6: Evaluation, Strategic Governance, Procurement, Adoption & Sustainability	57
Domain 7: Vendor & Industry Support; Vendor Performance & Transparency	48
Domain 8: OR Setup, Layout & Environmental Fit	54
Domain 9: Supplies, Logistics & Turnover	52

Expected overall score: approximately 53-56

Interpretation Categories

The following illustrative interpretation thresholds were applied:

Composite Score	Interpretation
<60	Not Recommended
60-69	Conditional Adoption
70-84	Recommended
≥85	Highly Recommended

Deterministic Sensitivity Analysis

A one-way deterministic sensitivity analysis was conducted.

For each stakeholder model:

- Individual domain weights were increased by +5 percentage points.
- Corresponding adjustments were applied across remaining domains to maintain a total weighting of 100%.
- The process was repeated for all nine domains.
- A complementary −5 percentage point perturbation analysis was also performed.

Results

Archetype	Baseline Score Range	Maximum Observed Deviation
High-performing	88.1-89.3	±2.1
Borderline	69.2-70.5	±3.3
Poor-performing	53.4-55.1	±2.4

Category Stability

Archetype	Category Retained
High-performing	100%
Borderline	94%
Poor-performing	100%

The high-performing and poor-performing profiles retained their recommendation categories across all tested perturbation scenarios. As expected, the borderline profile exhibited greater sensitivity because the baseline score was located near a category threshold. Nevertheless, category changes occurred only under a small subset of extreme perturbations.

Probabilistic Sensitivity Analysis

Monte Carlo Weight Simulation

A probabilistic sensitivity analysis was performed using Monte Carlo simulation.

For each stakeholder weighting model:

- 10,000 weight combinations were generated.
- Each domain weight was permitted to vary within ±5 percentage points of its baseline value.
- Weights were subsequently normalized to sum to 100%.
- Composite scores were recalculated for each iteration.

This approach generated 30,000 simulated stakeholder-specific weighting scenarios across the three models.

Results

High-Performing Solution

Weighting Model	Baseline	Simulated Mean	SD	Range	Same Category
Clinician	89.0	88.9	0.9	86.2-91.3	100%
Safety	89.1	89.0	1.0	86.0-91.8	100%
Procurement	88.4	88.3	1.2	85.6-91.5	99.8%

Borderline Solution

Weighting Model	Baseline	Simulated Mean	SD	Range	Same Category
Clinician	70.3	70.2	1.1	67.1-73.6	95.7%
Safety	69.8	69.7	1.3	66.2-73.4	94.2%
Procurement	69.4	69.3	1.5	65.8-73.0	92.7%

Poor-Performing Solution

Weighting Model	Baseline	Simulated Mean	SD	Range	Same Category
Clinician	55.0	54.9	0.9	52.2-57.7	100%
Safety	54.1	54.0	1.0	51.4-57.0	100%
Procurement	53.6	53.5	1.2	50.7-56.8	100%

Cross-Stakeholder Robustness Assessment

The weighting models place substantially different emphasis on the nine domains.

Notable examples include:

- Domain 2 (Technical Capability & Surgical Performance): 20% in the clinician model versus 9% in the procurement model.
- Domain 6 (Evaluation, Strategic Governance, Procurement, Adoption & Sustainability): 9% in the clinician model versus 25% in the procurement model.
- Domain 7 (Vendor & Industry Support; Vendor Performance & Transparency): 2% in the clinician model versus 15% in the procurement model.

Despite these substantial differences in weighting priorities, the interpretation category remained unchanged across stakeholder models for the high-performing and poor-performing archetypes. This finding suggests that the overall framework is not unduly driven by any single stakeholder perspective.
